# Autophagic Degradation of Misfolded Nuclear Receptor Co-repressor (NCoR) Is Linked to the Growth of Tumor Cells in HBX Positive Hepatocellular Carcinoma (HCC)

**DOI:** 10.3389/fonc.2019.01335

**Published:** 2019-12-03

**Authors:** Su Yin Tan, Sridevi Visvanathan, Radzi Abu Hassan, Matiullah Khan

**Affiliations:** ^1^Bio-Rad Laboratories, Singapore, Singapore; ^2^Departments of Biochemistry, AIMST University, Bedong, Malaysia; ^3^Clinical Research Center, Hospital Sultanah Bahiyah, Alor Setar, Malaysia; ^4^Department of Pathology, AIMST University, Bedong, Malaysia

**Keywords:** NCoR, misfolded protein, HBX, autophagy, HCC

## Abstract

Hepatitis B virus (HBV) is causally linked to hepatocellular injury and cell death, which are followed by hepatocellular carcinoma (HCC) after a long latent period. The HBV derived X protein (HBX) is the most potent carcinogenic factor for HCC, however, the molecular mechanism of HBX-induced transformation of hepatic cells in HCC is poorly understood. We have shown that nuclear receptor co-repressor (NCoR) is essential for the spatial repression of global transcription by the promyelocytic leukemia oncogenic domains (PODs), a frequent target of viral oncoproteins like HBX and that disintegration of PODs due to misfolded conformation dependent loss (MCDL) of NCoR is linked to promyelocytic and monocytic acute myeloid leukemia (AML). Given the key role of NCoR in cellular homeostasis across various tissue subtypes, we hypothesized that HBX-induced MCDL of NCoR might be linked to HCC through similar mechanism. Based on this hypothesis, the conformation of NCoR in HCC derived tumor cells and primary human tissue sections were analyzed and a selective MCDL of NCoR in HBX positive HCC cells was identified. HBX triggered the misfolding of NCoR through ubiquitination, followed by its degradation by autophagy, thus suggesting a cross talk between ubiquitin proteasome system (UPS) and autophagy lysosomal pathway (ALP) in MCDL of NCoR in HBX positive HCC cells. SiRNA-induced NCoR ablation selectively impaired the growth and survival of HBX positive HCC cells, suggesting a role of MCDL in the growth and survival of HBX positive HCC cells. These finding identify a possible crosstalk between UPS and ALP in the misfolding and loss of NCoR in HBX positive HCC cells and suggest a role of autophagic recycling of misfolded NCoR in the activation of oncogenic metabolic signaling in HCC. The misfolded NCoR reported in this study represents a novel conformation based molecular target which could be valuable in the design and development of tumor cell specific diagnostic and therapeutic approach for HBX positive HCC.

## Introduction

Primary liver cancer is one of the most lethal human malignancies for which no effective treatment is available. Liver cancer consists of several histologically different primary hepatic malignancies, but hepatocellular carcinoma (HCC) is by far the most common subtype, accounting for up to 85% of all cases of liver cancer (1). More than 80% of all HCCs are attributed to infection with either hepatitis B (HBV) or C (HCV) virus or both (2). Hepatitis B virus (HBV) is a 3.2 kb long double stranded DNA virus that primarily trigger inflammatory response in liver parenchyma, leading to hepatocellular injury and cell death, which are followed by HCC after a long latent period. However, it is not clear how a highly cytotoxic virus like HBV could implant the seed of transformation in the surviving hepatocytes which would eventually lead to HCC. Among all the HBV derived pathogenic proteins, the 16.5 kDa HBV X (HBX) protein is considered to be the most potent carcinogenic factor for HCC (3). *In vivo* studies have shown that HBX can directly transactivate a large number of promoters involved in inflammation and cell proliferation (4, 5). This mechanism allows HBV to undergo favorable alteration in the cellular microenvironment for further viral replication (4). In virus infected host liver cells, HBX can induce variety of responses, such as genotoxic stress, transcription modulation, protein degradation, and apoptosis (5). HBX has since been proposed to be strongly correlated to the development and progression of HCC, however, its exact role in the transformation of hepatocytes has not been fully elucidated.

PML oncogenic domains (PODs), which play important role in the cellular defense mechanism against pathogenic viruses, are known to be a frequent target of various carcinogenic factors, including pathogenic viral oncoproteins (6–8). Functionally, PODs are regarded as global repressor domains essential for the suppression of unwanted transcription, including viral transcription and replication (9). The repressive function of PODs is largely mediated by a global transcriptional co-repressor known as nuclear receptor co-repressor (NCoR), which is recruited to PODs for short and long term repression of target genes involve in cellular hemostasis (10–12). NCoR was originally identified as a co-repressor of un-liganded nuclear hormone receptors and the sequence specific transcriptional factor Mad (10, 13, 14). We have previously shown that PML-RAR, the fusion oncoprotein linked to the pathogenesis of promyelocytic acute myeloid leukemia (AML), can induce a characteristic ubiquitin-proteasome system (UPS) mediated misfolding of NCoR protein, which ultimately contributed to the disintegration of PODs in promyelocytic AML (15, 16). Retinoic acid, a potent inducer of differentiation of promyelocytic AML cells, abrogated NCoR misfolding and reorganized the PODs in promyelocytic AML cells, thus suggesting an important role of PODs in cellular defense against malignant transformation (17). These finding also suggested an important role of NCoR in the structural and functional integrity of PODs, which oncogenic virus like HBV must overcome to promote cellular transformation. The misfolded conformation dependent loss (MCDL) of NCoR initially identified in promyelocytic AML was later found to be involved in the pathogenesis of monocytic AML and non-small cell lung cancer (NSCLC), suggesting that MCDL might act as fundamental oncogenic mechanism to activate oncogenic metabolic pathway linked to the growth and survival of tumor cells in various tissue subtypes (18–22). Therefore, depending on the cell type involved, the tumor cell specific degradation of misfolded NCoR may promote uncontrolled growth and transformation by ectopic reactivation of cellular stemness in relatively matured myeloid cells of monocytic AML (21) while it could activate pro-survival oncogenic signaling such as UPR and autophagy in nutrient depleted solid tumor microenvironment of NSCLC (22).

Autophagy is a catabolic process that plays a housekeeping role by removing the misfolded or aggregated proteins from eukaryotic cells and by clearing damaged organelles such as mitochondria, endoplasmic reticulum and peroxisomes, as well as eliminating intracellular pathogens from host cells (23, 24). The process of autophagy begins with the formation of an isolation membrane or phagophore, which gradually expands to engulf the damaged cytoplasmic material destined to be degraded (the cargo), and sequesters the cargo in a double membrane vesicles known as autophagosome (25). The cargo loaded autophagosome then fuses with lysosomes, thus forming the autophagolysosome, which facilitates degradation of autophagosomal contents by lysosomal enzymes. Lysosomal transporters then export the released amino acids and other by-products of degradation back to the cytoplasm, where they are re-used for building macromolecules that are used as fuels for cellular metabolism (26). Thus, the autophagy mediated degradation of cargo is regarded as cellular “recycling factory” that enables cells to generate extra energy essential for sustaining cell growth and viability in nutrient depleted cellular microenvironments (27, 28). Based on these evidences, we argued that autophagic degradation of misfolded NCoR might also contribute to the growth and survival of tumor cells through restoring the energy balance in nutrient depleted cellular microenvironment widely prevalent in solid tumors like lung and liver cancer. Given the nearly identical effect of PML-RAR and pathogenic viral proteins on the structural and functional integrity of PODs, we hypothesized the HBX-induced transformation of liver cells might be the consequence of a prosurvival mechanism selectively activated by misfolded NCoR protein in HBX positives HCC cells. Here, we report that HBX promotes misfolding of NCoR protein through UPS and that degradation of misfolded NCoR through autophagy lysosomal pathway (ALP) is linked to the growth and survival of tumor cells in HBX positive HCC cells.

## Results

### Post-transcriptional Loss of NCoR Protein in HBX Positive HCC Cells

**Figure 1:**
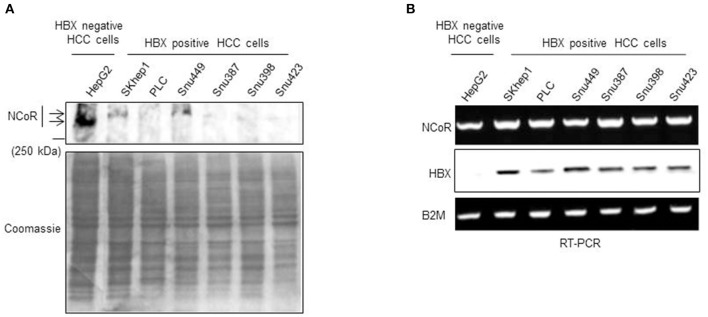
Post-transcriptional loss of NCoR protein in HBX positive HCC cells. **(A)** Level of full length NCoR protein in the whole cell extracts of HepG2 (HBX negative HCC cells) and multiple HBX positive HCC cells (SKHep1, PLC, Snu 449, Snu387, Snu398, and Snu423) was determined through western blotting. Arrows indicate the relative mobility of NCoR protein in HBX negative and positive HCC cells. As loading control, an aliquot of same extracts was resolved by SDS-PAGE and stained with coomassie blue dye. **(B)** Levels of NCoR and HBX transcripts in HBX negative HCC cells (HepG2) and HBX positive HCC cells (SKHep1, PLC, Snu 449, Snu387, Snu398, and Snu423) were determined by RT-PCR analysis. Level of β2M was determined as control.

Previously, we have shown that heterogeneous carcinogenic factors such as PML-RAR, MLL1-AF9 and nicotine can trigger misfolded conformation dependent loss (MCDL) of NCoR protein in promyelocytic and monocytic AML and NSCLC respectively (18–22). The MCDL of NCoR protein in these tumor cells was distinctly characterized by aberrant post-translational modification and destabilization of native state of NCoR protein, as well as its aberrant cytosolic retention and degradation by cellular protein quality control mechanism such as UPR and autophagy (18–22). To investigate whether misfolded conformation dependent loss of NCoR also plays any role in the pathogenesis of HCC, the level of intact NCoR protein in commercially available HBX positive HCC cells (SKHep1, PLC, Snu449, Snu387, Snu398, and Snu423) and HBX negative HCC cells (HepG2) was determined by western blotting assay. As shown in Figure 1A, the level of intact NCoR protein (270 kDa) in all HBX positive HCC cells (SKHep1, PLC, Snu449, Snu387, Snu398, and Snu423) was significantly lower when compared to its level in HBX negative cell HepG2. Among the six HBX positive HCC cells; PLC, Snu387, Snu398, and Snu423 displayed complete lack of intact NCoR protein while in SKHep1 and Snu449 cells, a faint band of intact NCoR was visible (Figure 1A). However, when compared to HBX negative HCC cell HepG2, NCoR level in all HBX positive HCC cells was significantly lower (Figure 1A). Moreover, the faint intact NCoR band observed in HBX positive HCC cells SKHep1 and Snu449 appeared to be slightly higher in molecular weight when compared to NCoR of HepG2 cells, the HBX negative HCC cells; suggesting that NCoR found in HBX positive HCC cells might have undergone some sort of post-translational modification (Figure 1A). Next, to determine if the lack of NCoR protein in HBX positive HCC cells was a post-transcriptional event, level of NCoR transcripts in all HBX positive HCC cells was compared to its level in HBX negative HCC cells HepG2 by RT-PCR. As shown in Figure 1B, the level of NCoR transcripts in all HBX positive HCC cells was more or less similar to that of HBX negative HCC cells HepG2, thus suggesting that lack of NCoR protein in HBX positive HCC cells was most likely a post-transcriptional event such as destabilization of native state and degradation of protein rather than down regulation of NCoR transcript (Figure 1B). The inverse correlation between NCoR protein and HBX transcript in HBX positive HCC cells; SKHep1, PLC, Snu 449, Snu387, Snu398, and Snu423 suggested that HBX might have a role in the loss of NCoR protein in these cells (Figure 1B).

### HBX Promotes Degradation of NCoR Protein

**Figure 2:**
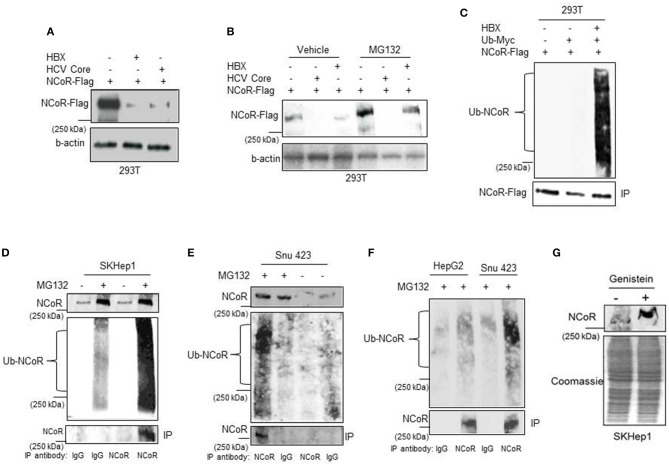
HBX promotes degradation of NCoR protein. **(A)** HBX promotes degradation of NCoR protein. Level of intact NCoR-Flag in whole cell extract of 293T cells transfected with Flag tagged NCoR and HBX or HCV plasmids was determined in western blotting assay using anti-Flag antibody. **(B)** Level of intact NCoR-Flag in whole cell extract of 293T cells transfected with NCoR-Flag and HBX or HCV core plasmids and treated with vehicle or 10 μM MG132 for 12 h was determined in western blotting assay with anti-Flag antibody. **(C)** HBX promotes NCoR ubiquitination. NCoR-Flag was immunoprecipitated with anti-Flag antibody from the whole cell extracts of 293T cells transfected with various combinations of plasmids as mentioned on top of each lane and stained with Myc antibody. A significantly higher level of ubiquitinated NCoR was observed in 293T cells co-transfected with HBX plasmid (upper panel). The membrane was re-probed with anti-Flag antibody to determine the amount of immunoprecipitated (IP) NCoR-Flag (lower panel). **(D–F)** Endogenous NCoR protein from SKHep1 **(D)**, Snu423 **(E)**, or HepG2 **(F)** cells treated with vehicle or MG132 as indicated on top of each lane was immunoprecipitated with anti-NCoR antibody and level of ubiquitinated (Ub) NCoR protein in each immunoprecipitate was determined by western blotting assay with anti-Ub antibody (middle panel). The membrane was then re-probed with anti-NCoR antibody to quantify the amount of immunoprecipitated (IP) NCoR protein (lower panel). Level of NCoR protein (input) in each starting sample was determined in western blotting assay with anti-NCoR antibody (upper panel). **(G)** Genistein stabilizes the NCoR protein. Level of intact NCoR protein in whole cells extract of SKHep1 cells treated with vehicle or genistein (25 μM) for 48 h was determined in western blotting assay using anti-NCoR antibody.

Next to confirm that HBX was involved in the loss or degradation of NCoR protein, level of intact NCoR protein in 293T cells transfected with Flag tagged NCoR (NCoR-Flag) and HBX or HCV core plasmids was determined through western blotting assay. The level of full length NCoR-Flag protein was significantly lower in 293T cells co-transfected with HBX or HCV expression plasmids, suggesting a role of HBX/HCV in the loss of NCoR-Flag protein (Figure 2A). We have previously shown that post-translational modification of NCoR through UPS was linked to its misfolding and subsequent loss in promyelocytic AML (16). Consistent with those finding, the unstable NCoR which was subjected to loss in HBX positive HCC cells also appeared to be post-translationally modified as it displayed slower migration in SDS-PAGE when compared to NCoR of HBX negative HCC cell HepG2 (Figure 1A). Next, to test whether HBX-induced loss of NCoR in HCC positive cells was ubiquitin-proteasome mediated, effect of MG132, a selective inhibitor of proteasome function, on HBX-induced NCoR loss was determined in 293T cells transfected with Flag tagged NCoR and HBX or HCV core plasmids. The HBX-induced NCoR loss was only partially blocked by MG132, while MG132 had no effect on HCV-induced NCoR loss, suggesting the involvement of some other proteolytic pathway in HBX/HCV-induced loss of NCoR protein (Figure 2B).

Before their clearance by UPS, most substrate proteins are tagged with ubiquitin so that they can be recognized by proteasome. Therefore, to test whether HBX-induced NCoR degradation is preceded by its ubiquitination, level of ubiquitinated NCoR in MG132 treated 293T cells co-transfected with NCoR-Flag and ubiquitin-myc plasmids was determined through *in-vitro* ubiquitin assay. As shown in the results of ubiquitin assay, the level of ubiquitinated NCoR (Ub-NCoR) in HBX co-transfected 293T cells was significantly higher when compared to its level in cells lacking HBX (Figure 2C, upper panel). Ubiquitinated NCoR was not detected in 293T cells transfected with NCoR-Flag expression plasmid alone (Figure 2C, upper panel). The amount of immunoprecipitated (IP) NCoR-Flag protein was more or less similar in all experimental setups (Figure 2C, lower panel) despite the significant difference in the level of ubiquitinated NCoR. To investigate whether misfolded NCoR was also ubiquitinated in HBX positive HCC cells which exhibited NCoR loss in western blooting assay (Figure 1A), level of ubiquitin conjugated NCoR in HBX positive SKhep1 or Snu423 cells treated with vehicle or MG132 was determined by immunoprecipitation assay. In this assay, NCoR protein from the whole cell extracts of SKhep1 or Snu423 cells treated with vehicle or MG132 was first immunoprecipitated with anti-NCoR antibody and then probed with anti-Ub antibody. In MG132 treated SKhep1 or Snu423 cells, level of ubiquitinated NCoR was significantly higher when compared to its level in vehicle treated cells, suggesting that a major portion of NCoR protein in MG132 treated SKhep1 or Snu423 cells was ubiquitin modified and possibly misfolded (Figures 2D,E). Interestingly, NCoR immunoprecipitated from HBX negative HepG2 cells did not exhibit much ubiquitination when compared to NCoR from HBX positive Snu423 cells (Figure 2F). Together, these finding suggested a role of HBX in the ubiquitination of NCoR in transfected 293T cells as well as in HBX positive HCC cells SKHep1 and SNU423. However, despite being tagged with Ub, the misfolded NCoR was apparently not degraded by proteasome as MG132 failed to block the HBX/HCV-induced loss of NCoR protein efficiently (Figure 2A). Next, to test if HBX-induced NCoR loss was indeed due to its misfolding, effect of genistein, an inhibitor of NCoR misfolding previously identified by us (17), on the level of NCoR protein was determined in SKHep1 cells. As expected, genistein at 25 μM concentration, effectively blocked the loss of NCoR protein, leading to its stabilization in SKHep1 cells as demonstrated by the appearance of intact NCoR protein after genistein treatment (Figure 2G). These finding collectively suggested that NCoR protein that was subjected to degradation by HBX in HBX positive HCC cells was ubiquitin modified and was most likely misfolded.

### HBX Promotes Cytosolic Localization of NCoR Protein

**Figure 3:**
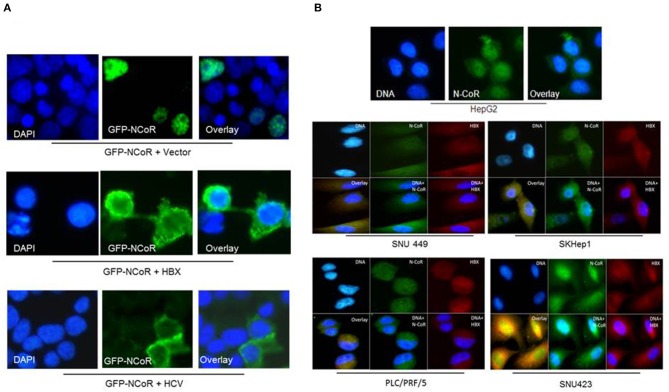
HBX promotes cytosolic retention of NCoR protein. **(A)** Sub-cellular distribution of GFP-NCoR (green fluorescence NCoR) co-expressed in 293T cells with empty vector, HBX or HCV plasmids were determined by immunofluorescence staining. When expressed alone, GFP-NCoR was mainly localized in the nucleus of 293T cells (topmost panels). In 293T cells co-expressing GFP-NCoR and the HBX or HCV protein, a significant portion of GFP-NCoR was found to be localized in the cytosole of 293T cells. **(B)** Sub-cellular distribution of endogenous NCoR in HBX negative HCC cells HepG2 (topmost panel) and several HBX positive HCC cells was determined by immunofluorescence assay using anti-NCoR and HBX antibodies, followed by staining with fluorescence labeled secondary antibodies. Signals were acquired through fluorescence microscopy. Green signal represents NCoR distribution while HBX is represented by red signal. Nucleus was stained with DAPI and is represented by blue signals. In HepG2 cells, majority of NCoR signal was detected in the nucleus overlapping the DAPI signal. However, in HBX positive cells, a significant portion of NCoR signal was detected in the cytosol where it overlapped with HBX signal (yellow signals). Original magnification, X 400.

We have previously reported that intact and stable NCoR protein present in normal cells is localized in the nucleus while the unstable and misfolded NCoR found in the transformed cells are mostly targeted to the cytosol (18–22), thus suggesting that aberrant cytosolic localization is an important hallmark of misfolded NCoR protein. Therefore, to further characterize the role of HBX in the misfolding of NCoR protein in HCC cells, the subcellular distribution of GFP tagged NCoR co-expressed with HBX or HCV in 293T cells was determined through immunofluorescence assay. As expected, the GFP tagged NCoR was preferentially localized to the cytosol of 293T cells when co-expressed with HBX or HCV (Figure 3A, lower panels), while NCoR co-expressed with the empty vector was localized mainly in the nucleus (Figure 3A, topmost panel). To validate the above finding in HCC cells, subcellular distribution of endogenous NCoR in HBX positive and negative HCC cells was determined. Consistent with the observation in 293T cells, a major portion of NCoR protein in multiple HBX positive HCC cells was found to be localized in the cytosol (Figure 3B, lower panels), while in HBX negative HepG2 cells, NCoR displayed a predominantly nuclear localization (Figure 3B, topmost panel). Moreover, a significant portion of NCoR signal in the cytosol of HBX positive HCC cells overlapped with the signal of HBX protein (Figure 3B, overlay). These finding collectively suggested a possible role of HBX in the cytosolic retention of misfolded NCoR protein in HBX positive HCC cells.

### NCoR Displays Cytosolic Localization in Primary Human HCC Tissue Sections

**Figure 4:**
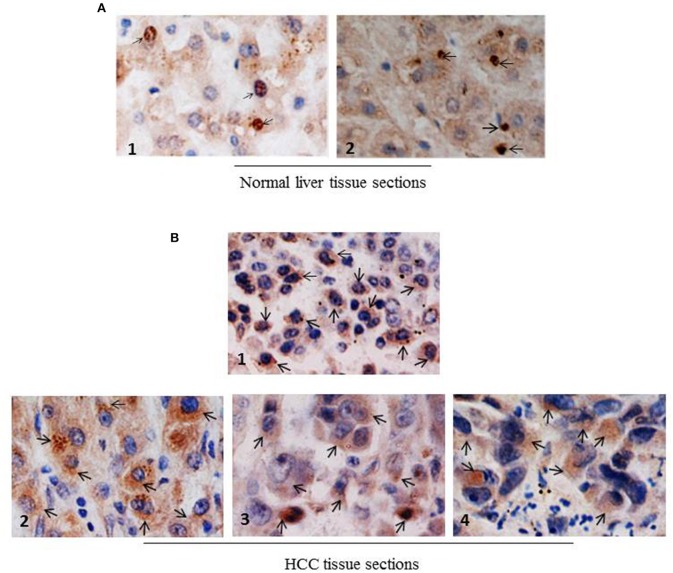
NCoR displays cytosolic localization in primary human HCC tissue sections. **(A)** Subcellular distribution of NCoR in normal human liver tissue sections was determined by immunohistochemistry using NCoR antibody. The brown stains marked by arrow represent the NCoR protein while the blue stains indicate the nucleus. **(B)** Subcellular distribution of NCoR in four independent (panels 1–4) primary human HCC tissue sections was determined by immunohistochemistry using NCoR antibody. The brown stains marked by arrow represent the NCoR protein while the blue stains indicate the nucleus. The NCoR displayed a characteristic cytosolic distribution in HBV positive HCC tissue sections (panels 2–4) while in HBV negative HCC tissue, NCoR signal mostly overlapped with the nucleus (panel 1). Original magnification, X 400.

Next, to investigate whether NCoR was also misfolded in primary human liver cancer tissue, subcellular distribution of NCoR in two normal liver and four histologically confirmed HCC tissue sections with known HBV status was determined by immunohistochemistry (IHC) using anti-NCoR antibody. Consistent with the findings of HBX positive HCC cell lines, a major portion of NCoR protein (marked by arrow) in three HBV positive HCC tissue sections was found in the cytosol (Figure 4B, panels 2–4). Majority of tumor cells within the captured field of these three (panels 2–4) slides displayed the characteristic cytosolic distribution of NCoR marked by brown stains surrounding the darkly stained nucleus. However, as seen in HBX negative HCC cell HepG2 (Figure 3B, topmost panel), the NCoR signals in most of the tumor cells in HBV negative HCC tissue section largely overlapped with the nucleus and the perinuclear region with some degree of extension into the cytosole (Figure 4B, panel 1), while the NCoR signals were predominantly visible in the nucleus of normal liver tissue section (Figure 4A, panels 1, 2). Further examination of NCoR in additional view fields of slides 2, 3, and 4 exhibited the characteristic cytoplasmic distribution of NCoR in around 50–60% cells.

### The Degradation of Misfolded NCoR in HBX Positive HCC Cells Is Mediated by Autophagy Lysosomal Pathway

**Figure 5:**
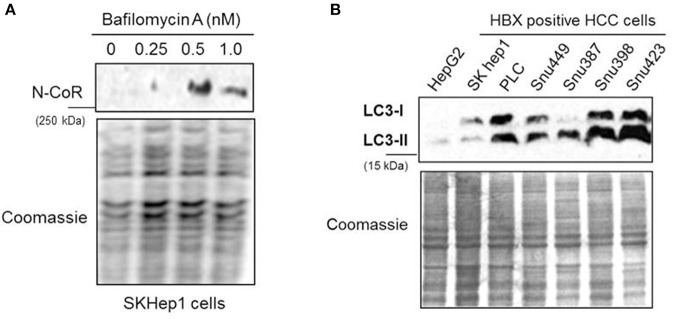
NCoR degradation in HBX positive HCC cells is mediated by autophagy. **(A)** Bafilomycin A1 (BA-1) blocked the loss of NCoR protein in SKHep1 cells. Level of full length NCoR protein in SKHep1 cells treated with BA-1 for 72 h was determined by western blotting assay. BA-1 effectively restored NCoR protein level at 0.5 nM concentration. **(B)** Level of LC3-I/II in HCC cells (upper panel) were determined through western blotting assay performed with anti LC3 antibody. The higher level of LC3-II seen in the HBX positive cells indicated an activated level of autophagy. Coomassie staining (lower panel) served as an experimental control.

The partial abrogation of NCoR loss by MG132, the selective inhibitor of proteasome function (Figure 2B), suggested the involvement of some other proteolytic pathway in the loss of NCoR protein in HBX positive HCC cells. We have previously reported that post-transcriptional loss of misfolded NCoR in non-small cell lung cancer cells was linked to autophagy n (22). Therefore, to test whether autophagy played any role in the degradation of misfolded NCoR in HBX positive HCC cells, level of full length NCoR protein in SKHep1 cells treated in a dose dependent manner with bafilomycin A1 (BA-1), a selective inhibitor of autophagy, was determined by western blotting assay. BA-1 effectively stabilized the NCoR protein in SKHep1 cells at 0.5 nM concentration and above, thus suggesting a role of autophagy in the loss of NCoR protein in HBX positive HCC cells (Figure 5A). To further confirm the role of autophagy in the loss of NCoR protein in HBX positive HCC cells, the level of LC3II:LC3I proteins essential for the formation of autophaosome, the double membrane vesicle which delivers misfolded proteins to the lysosomes for degradation during autophagy, was determined (25). LC3-II, the processed form of LC3, has a reported molecular weight of 14 kDa while the unprocessed LC3-I is a 16 kDa protein. As shown in Figure 5B, level of LC3II:LC3I was significantly higher in almost all the HBX positive HCC cells when compared to their level in HBX negative cells HepG2, suggesting a selective up-regulation of autophagy in HBX positive HCC cells.

### HBX and NCoR Could Induce Active Autophagosome Formation

**Figure 6:**
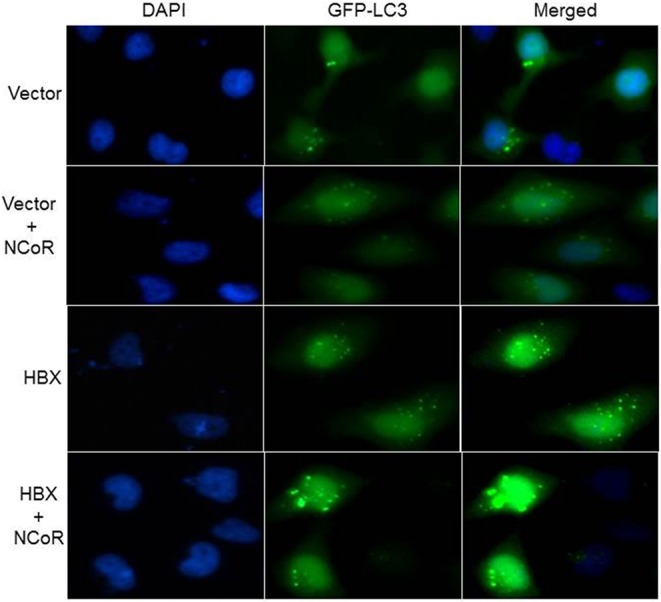
HBX induced autophagy is mediated by misfolded NCoR protein. HeLa cells were transfected with the GFP-LC3 plasmid, together with various combinations of plasmids as shown on the left of each row. Sub-cellular localization as well as intensity of LC3 (green) signal was determined by fluorescence microscopy. Punctuated dot like structures of GFP-LC3 appeared to be more prominent in the presence of HBX and NCoR. Original magnification, X 400.

**Figure 7:**
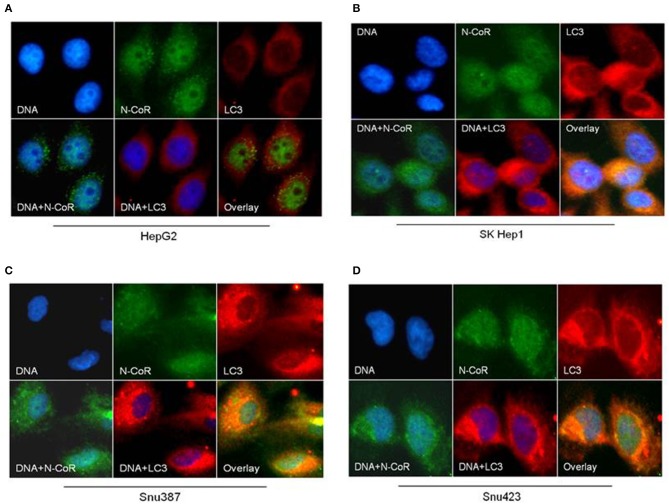
NCoR is co-localized with LC3 in HBX positive HCC cells. HCC cells were fixed and stained for endogenous NCoR (green) and LC3 (red) with NCoR and LC3 antibodies respectively. DNA was stained with DAPI (blue). Significant co-localization of NCoR (green) and LC3 (red) signal was observed as orange-yellow signals in the HBX positive HCC cells **(B–D)**, while no co-localization was observed in HepG2 cells **(A)**. Original magnification, X 400.

Beside the level and ratio of LC3II:LC3I proteins, active intracellular autophagosomes could be characterized by characteristic expansion and punctuated distribution of LC3 proteins detected by immunofluorescence assay in the cystole of cells undergoing autophagy. Therefore, to test whether HBX could induce active autophagosome formation and to what extend this autophagosome formation could be influenced by co-expression of NCoR protein, number of punctuated LC3 in 293T cells transfected with GFP tagged LC3 along with HBX or NCoR plasmid was determined through immunofluorescence assay. As shown in Figure 6, GFP-LC3 exhibited fewer punctuated distribution in 293T cells when it was expressed alone or in combination with HBX or NCoR expression plasmids. However, when GFP-LC3 was co-expressed with both HBX and NCoR plasmids, the number and size of punctuated GFP-LC3 signals was expanded significantly, suggesting a role of HBX induced misfolded NCoR in the formation of punctuated distribution of LC3 in 293T cells (Figure 6). The punctuated distributions of LC3 protein, which showed significant co-localization with NCoR protein, were also more prominent in all HBX positive HCC cells (Figures 7B–D) when compared to HBX negative cells HepG2 (Figure 7A). We also observed partial co-localization of NCoR (green signals) with LC3 (red signals) within the autophagosomes as indicated by the speckled orange-yellow signals (Figures 7B–D, overlay). In contrast, the intensity of red LC3 signal was significantly lower in HepG2 cells with little overlapping with NCoR signal (Figure 7A). These finding collectively suggested that HBX-induced misfolding of NCoR might be linked to the selective activation of autophagy in HBX positive HCC cells.

### Autophagic Degradation of Misfolded NCoR Is Linked to the Growth and Survival of HBX Positive HCC Cells

**Figure 8:**
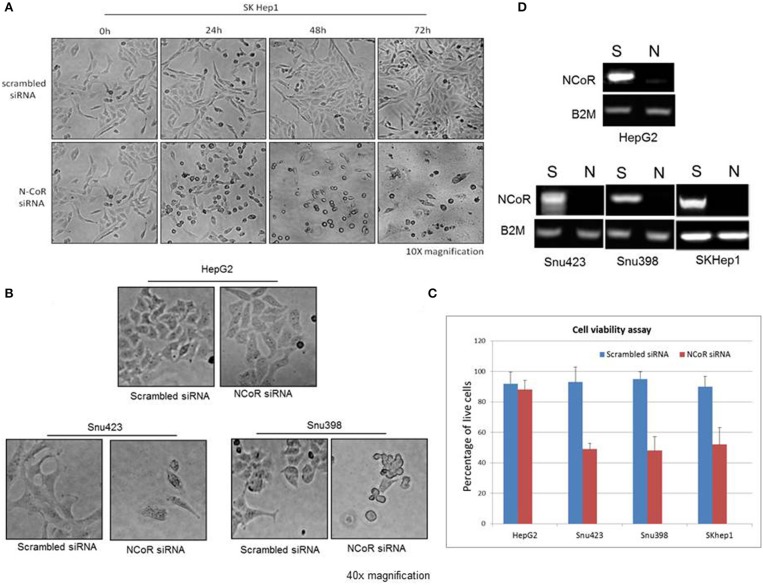
Misfolded NCoR is linked to the growth and survival of HBX positive HCC cells. **(A)** The growth and survival of HBX positive HCC cells SKhep1 exposed to scrambled or anti-NCoR siRNA for 0–72 h was determined by morphological analysis. **(B,C)** The survival of HBX negative (HepG2) and positive (SNU423 and SNU398) HCC cells exposed to scrambled or anti-NCoR siRNA for 48 h was determined by morphological analysis **(B)** and trypan blue dye exclusion tests performed in triplicate **(C)**. **(D)** NCoR knockdown efficiency for each sample was determined by RT-PCR.

Recently, metabolic homeostasis mediated by autophagy has emerged as an important prosurvival mechanism in tumor cells during nutrient stress in some cancer, including ductal pancreatic carcinoma and lung cancer (22, 29–34). Cargo degraded through autophagy was linked to the production of metabolic intermediates that sustained the growth of tumor cells through the activation of pro survival oncogenic metabolic pathways (33, 35, 36). Based on these findings, we thought that autophagy-mediated degradation of misfolded NCoR may also confer pro-survival advantage to HCC cells during nutrient stress commonly encountered by tumor cells in solid tumor microenvironment like HCC. To investigate if autophagic degradation of misfolded NCoR was involved in sustaining the growth and survival of HBX positive HCC cells, the growth and survival of *NCoR* ablated HCC cells was determined by morphological analysis. SiRNA-induced *NCoR* ablation significantly impaired the growth and survival of SKHep1, the HBX positive HCC cells, in a time dependent manner, the maximum effect being apparent within 48 h of culture (Figure 8A). Similarly, HBX positive HCC cells Snu423 or Snu398 treated with anti-NCoR siRNA for 48 h also displayed significant reduction in growth (Figure 8B) along with 40–45% reduction in the number of live cells when compared to HBX negative cells HepG2 (Figures 8C,D), suggesting that misfolded NCoR was somehow linked to the growth and survival of HBX positive HCC cells. These finding collectively suggested that the HBX-induced autophagic degradation of misfolded NCoR protein was most likely linked to the growth and survival of tumor cells in HBX positive HCC.

## Discussion

A link between malignant growth and transformation of tumor cells and the degradation of misfolded NCoR protein was first established when we demonstrated that PML-RARα, the fusion oncogene linked to promyelocytic AML, could act as an E3 ligase and trigger degradation of misfolded NCoR protein through UPS in promyelocytic AML (16). Subsequently, MLL1-AF9, another fusion oncoprotein linked to monocytic AML, was also found to induce misfolded conformation dependent loss of NCoR through similar mechanism (19–21). The findings reported in this manuscript are consistent with those finding and suggest that HBX can also create a PML-RARα or MLL1-AF9 like effect on the conformation of NCoR protein through aberrant post-translational modification and transform the liver cells through similar oncogenic gain of function mechanism as observed in promyelocytic and monocytic AML. As shown by us previously, NCoR is a structural and functional component of PML oncogenic domains (PODs) which are essential for the spatial and temporal repression of growth promoting genes during cellular differentiation and hemostasis (11, 12). Functionally, PODs are regarded as global repressor domains which are involved in the suppression of unwanted transcription, including viral transcription and replication, and therefore, they might be a target of pathogenic viral oncoproteins like HBX and HCV core proteins. As such, transcriptional repression mediated by the natively folded NCoR located in the PODs of liver cells may play important role in the cellular defense against hepatotropic virus like HBV, and that HBX-induced misfolding of NCoR protein and disintegration PODs may contribute to the transformation of hepatocytes through the de-repression of transforming viral as well as host oncogenes essential for the activation of oncogenic metabolic signaling linked to ALP. Although, the exact molecular mechanism underlying the oncogenic potential of HBX is still unclear; however, based on our data presented in this manuscript, it can be argued that HBX binds to NCoR and trigger its ubiquitin mediated misfolding and loss of function, ultimately leading to the de-repression of transforming viral and host oncogenes. The exact role of HBX in the ubiquitination of misfolded NCoR protein in HBX positive HCC cells remains unclear but based on our previous findings in promyelocytic AML where PML-RARα acted as an E3 ligase for the misfolded NCoR protein, a role of HBX as E3 ligase for misfolded NCoR in HBX positive HCC cells can't be rule out (16). It is also likely that HBX, being a misfolded protein itself, may simply associate with NCoR after its transcription during HBV infection and thus facilitate the change in NCoR conformation through ubiquitination by a yet to be characterized E3 ligase. Despite being heavily modified by ubiquitin, the misfolded NCoR was not apparently degraded by UPS as MG132 failed to block its loss. This apparent failure of clearance of ubiquitin modified NCoR by UPS was also observed previously by us in promyelocytic AML, which led to UPR-induced apoptosis (15, 16). The failure of misfolded NCoR clearance by UPS could be an outcome of direct inhibition of the proteasome function by misfolded NCoR as intracellular accumulation of misfolded proteins are known to inhibit proteasome function (37).

**Figure 9:**
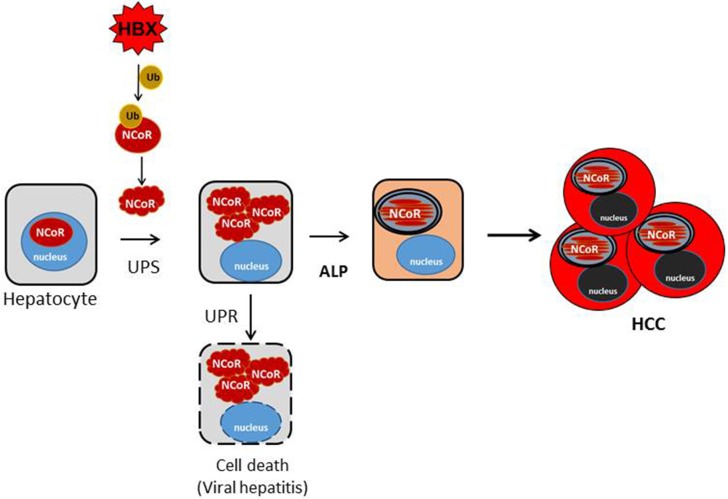
Schematic representation of the role of misfolded NCoR in the growth and survival of HBX positive HCC cells. HBX-induced ubiquitination of NCoR protein leads to the misfolding and cytosolic retention of NCoR protein in HBX positive HCC cells. Cytosolic accumulation of misfolded NCoR may initially trigger hepatic cell death though the UPR-induced apoptosis mechanism as seen during the initial stage of acute viral hepatitis caused by HBV infection. In some surviving HBV infected cells, loss of NCoR function due to misfolding may de-repress the NCoR target genes essential for the activation of ALP. After ALP activation, the autophagic degradation of misfolded NCoR could attenuate the UPR-induced apoptosis and activate the oncogenic metabolic signaling in the surviving hepatic cells, ultimately leading to HCC.

Most mammalian cells have robust protein quality control mechanisms to deal with the consequence of intracellular accumulation of misfolded proteins which are inherently cytotoxic in nature. The initial consequence of intracellular accumulation of misfolded proteins is the activation of unfolded protein response (UPR) which induces molecular chaperones to repair the conformation of misfolded proteins. However, if the conformation of misfolded protein cannot be corrected through UPR, then the misfolded proteins are tagged with ubiquitin for their subsequent identification and removal through the ubiquitin proteasome system (UPS). However, excess accumulation of intracellular misfolded proteins can also inhibit the function of proteasome, thus compromising the capacity of UPS to remove the misfolded protein load. Under the circumstance when both the UPR and UPS are unable to correct or remove the misfolded proteins, then these misfolded proteins are ultimately cleared through autophagy, which forms the pathogenic basis of some neurodegenerative diseases primarily caused by intracellular accumulation of misfolded proteins (38–40). Until recently, it was thought that UPS and autophagy are distinct pathways with little or no convergence at molecular level. However, HBX-mediated ubiquitination and misfolding of N-CoR and its subsequent degradation by autophagy as observed in our study suggest a significant overlap and crosstalk between UPS and autophagy in the degradation of misfolded NCoR where UPS tags the misfolded NCoR with ubiquitin for its recognition and clearance by autophagy. Although, evidence of crosstalk between UPS and autophagy have recently been documented in some neurodegenerative disorders like Alzheimer's (41) and Parkinson's diseases (42), this study is the first evidence suggesting that convergence of UPS and ALP can also occur in malignant disease like HCC. In HCC, the convergence appears to occur sequentially, first, the loss of NCoR function due to UPS mediated misfolding of NCoR may cause the de-repression of putative NCoR target genes, possibly a lysomosal protease involved in ALP pathway, eventually leading to the degradation of misfolded NCoR protein through autophagy (Figure 9).

Reports of autophagy being involved in the removal of misfolded proteins and in the alleviation of ER stress to non-cytotoxic level have been reported in Huntington disease (36), Alzheimer's disease (33) and Parkinson disease (38). These neurodegenerative diseases are distinctly characterized by extensive neuronal cell death triggered by misfolded protein accumulated as a consequence of impaired proteasome function. Hepatic cell injury and death during the early stage of viral hepatitis are also an immediate outcome of inflammation of liver parenchyma by HBV, which is followed by HCC after a long latent period. It is a paradox how HBV, a potentially cytotoxic virus, could induce transformation in hepatic cells undergoing apoptosis. It is likely that hepatic cell death during the early stage of viral hepatitis is triggered by the UPR-induced apoptosis mediated by intracellular accumulation of misfolded NCoR as shown by us previously in APL cells (15), while the autophagic degradation of misfolded N-CoR protein may facilitate the revival of surviving hepatic cells, which eventually could lead to the development of HCC after a long latent period. In this background, the initial load of misfolded N-CoR during the early stage of HBV infection might be degraded by UPS, however, as the intracellular load of misfolded NCoR protein would continue to increase, it could compromise the proteasome function, leading to further accumulation of misfolded NCoR protein and thus trigger the UPR-induced apoptosis of HBV infected hepatic cells (Tan and Khan, unpublished manuscript). In some surviving HBV infected cells however, loss of NCoR function due to misfolding may de-repress the putative NCoR target genes essential for the activation of ALP. Thus, the autophagic degradation of misfolded NCoR may promote transformation through the sequential activation of two oncogenic signaling pathways; first, the activation of cytoprotective UPR through alleviating the cytotoxic insult of misfolded NCoR protein; other by producing amino acid metabolites from the autophagic catabolism of misfolded N-CoR (Figure 9). Recently, energy derived from the autophagy-induced recycling of amino acid metabolites has been linked to the growth of malignant cells in nutrient depleted tumor microenvironment in solid tumors (33, 35, 36). Thus, the autophagy-induced degradation of misfolded NCoR reported in this study may represent a compensatory metabolic reprograming activated by HBX oncoprotein for sustaining the growth of HCC cells in nutrient deplete tumor microenvironment.

## Experimental Procedures

### Cell Lines, Cell Culture, and Reagents

The HCC cell-lines; HepG2, SK Hep1, PLC/Prf/5, Snu449, Snu387, Snu398, Snu423, and Snu475 used in this study were purchased from the American Type Culture Collection (Rockville, MD). All cells were cultured in Dulbecco's modified eagle medium (Sigma Aldrich, USA) supplemented with 10% fetal bovine serum (FBS) (HyClone, Waltham, USA), at 37°C in a 5% Co_2_ humidified atmosphere. Media was changed every 3–4 days and cells were passaged by trypsinization with 0.5% (w/v) trypsin and 0.2% (w/v) ethylenediaminetetraacetic acid (EDTA) disodium salt when they reached 80–90% confluency. 293T or HeLa cells were seeded at 200 × 10^4^ per 10-cm plate or 80 × 10^4^ cells per 6-cm plate or 20 × 10^4^ cells per well of a 6-well-plate 1 day prior to transfection. Approximately 18–22 h after seeding, transfection was carried out. Briefly, Fugene 6 (Roche, Mannheim, Germany) was first diluted in serum free medium for 5 min at room temperature and indicated plasmids were then added to diluted Fugene (DNA: Fugene = 1 μg: 3 μl). The mixture was incubated for further 15 min at room temperature. The final DNA-Fugene 6-serum free medium mixture was then added slowly in a drop wise manner to the cells. Cells were then incubated for at least 48 h before harvesting. pAct-NCoR-Flag consists of 2 tandem repeats of Flag sequence, linked in frame to the C terminus of mouse NCoR sequence and cloned into the vector at Nco1 and Xba1 sites. The NCoR (C-20, goat polyclonal) antibody used for the western blotting and immunoprecipitation assays was purchased from Santa Cruz Biotechnology (CA, USA) and utilized as described previously (16, 43).

### Primary Liver Tissue and Tissue Microarray

Anonymous human normal and malignant liver tissue sections of clinically and histologically diagnosed HCC patients were obtained from the Hospital Sultanah Bahiyah, Alor Setar, Kedah, Malaysia. This study has been approved by Malaysian medical research and ethics committee, the national medical research register (NMRR-16-1166-31533-IIR). The custom liver tissue microarray was purchased from US Biomax, Rockville, MD United States.

### Protein Extraction

Cells were collected after trypsinization and pelleted by centrifuging at 200 g for 5 min at 4°C. The resulting pellet was rinsed twice with ice-cold 1X PBS (pH 7.4) before centrifugation and the supernatant was then removed. Four pellet volume of 2X SDS sample buffer [250 mM Tris-HCl (pH6.8), 40% glycerol, 9.2% SDS, 0.01% bromophenol blue, 20% β-mercaptoethanol] was used to re-suspend cell pellet. Cells were then sonicated twice or thrice, 10 s each, on ice using the Branson Sonifier 150 with an output power of 5 W. The protein samples were then denatured by heating at 50°C for 10 min or 95°C for 5 min (43).

### Western Blotting

Protein samples were resolved on SDS-PAGE using the Bio-Rad Mini-Protean II system. Electrophoresis was carried out at constant current of 10 mA using cold 1X Laemli running buffer [25 mM Tris, 192 mM glycine, 0.1% (w/v) SDS] at 4°C. The proteins were then transferred onto Hybond-P Polyvinylidene Fluoride (PVDF) membrane (GE Healthcare, Uppsala, Sweden) using a wet transelectroblotting system (Bio-Rad Inc., England). The polyacrylamide gel was run at a constant current of 60 mA for 150 min at 4°C in 1X transfer buffer [48 mM Tris, 39 mM glycine, 0.037% (w/v) SDS and 10% (w/v) methanol (added just prior to use)]. After transfer, membranes were blocked in PBS-T (1X PBS containing 0.1% Tween 20) containing 5% milk [5% non-fat milk powder (Merck) in 1X PBS-T] for 1 h at room temperature or overnight at 4°C. After incubation in the appropriate primary antibody overnight at 4°C in the respective blocking buffers, the membrane was rinsed thrice with PBS-T for 10 min each to remove any unbound antibodies. HRP-conjugated secondary antibodies with the blocking buffer were then applied for 1 h at room temperature. The unbound secondary antibodies were removed by washing the membrane 5 times for 10 min each with PBS-T before the detection of immunoreactive bands by Western Lightning Chemiluminescence Reagent Plus (Perkin Elmer, CA, USA). The X-ray film was finally developed using a Konica Minolta SRX-101A film processor. After immunodetection, the bound primary and secondary antibodies were removed from the membrane, which then can be re-probed with a different antibody. The developed membrane was briefly washed with PBS-T to remove residual chemiluminescence reagent and immersed in stripping buffer [glycine-HCl (pH2), 1% (w/v) SDS] for 30 min with agitation. The membrane was rinsed with large volumes of PBS-T thrice for 10 min each at room temperature and subsequently blocked with respective blocking buffers. The membrane was then re-probed by a different antibody using the same protocol described above. For the inhibition of NCoR misfolding by genistein, SKhep1 cells treated with vehicle or 25 μM genistein was cultivated for 48 h in DMEM supplemented with 10% FBS and NCoR level in whole cell extract, prepared in 2X sample loading buffer by mild sonication, was determined (43).

### Ubiquitination and Immunoprecipitation Assay

Cell were harvested in 4X pellet volume of lysis buffer [10 mM Tris-HCl (pH8), 500 mM NaCl, 2% SDS], heated at 95°C for 10 min and diluted with dilution buffer (lysis buffer:dilution buffer = 1:9) [10 mM Tris-HCl (pH8), 500 mM NaCl, 1% Triton-X-100), sonicated mildly twice for 10 s on ice. The cell lysate was then incubated for 2.5 h at 4°C with rotation with 5 μg of either anti-Flag or anti-NCoR antibodies (Santa Cruz Biotechnology, CA, USA) and anti-mouse or anti-goat IgG antibodies (Santa Cruz Biotechnology, CA, USA) were used as controls. After pre-adsorption with protein G- Sepharose beads (GE Healthcare Bio-Sciences AB, Sweden) for 2 h at 4°C with rotation, the immunocomplexes were pulled down, added to 2X SDS sample buffer containing 20% β-mercaptoethanol and heated at 50°C for 10 min. The eluted proteins were then subjected to SDS-PAGE (43).

### Immunohistochemistry

The paraffin-embedded human liver cancer tissue sections were baked at 60°C to remove the wax and to improve adhesion before staining. Immunohistochemical staining of tissue was done using the goat ImmunoCruzTM Staining System (Santa Cruz Biotechnology, CA, USA) following the manufactures protocol. Briefly, sections were deparaffinized with histoclear, rehydrated in decreasing ethanol concentrations, and the antigens unmasking was done using 1 mM EDTA (pH8) at 95°C for 10 min. Endogenous peroxidase activity was quenched using hydrogen peroxide, followed by incubating the sections with 5 μg goat anti-NCoR antibody (C-20; Santa Cruz Biotechnology, CA, USA) (1:40) for 2 h at room temperature. The sections were then treated with HRP conjugated dextran polymer for 30 min and further incubated with DAB+substrate chromogen solution for 5–10 min. Sections were counterstained with Mayer's hematoxylin and mounted.

### Immunofluorescence and Confocal Microscopy

Cells were grown on coverslips in culture medium till 50–60% confluency before transfection or when 80–90% confluency reached for untreated cells. They were then fixed with 4% paraformaldehyde in PBS (freshly prepared and pre-warmed to 37°C) for 30 min at 37°C, washed thrice with 1XPBS before permeabilizing with 0.2% Triton X-100 in PBS on ice for 5 min. Alternatively, cells were fixed and permeabilized with methanol at −20°C for 7 min. After permeabilization, cells were washed thrice with 1XPBS and blocked with 5% bovine serum albumin (BSA) in PBS for 30 min. Cells were then incubated in primary antibodies at their appropriate dilutions for 2 h at room temperature and subsequently washed with PBS thrice, 5 min each followed by incubation with the appropriate Alexa Fluor secondary antibodies (Invitrogen, Carlsbad, CA, USA) with 1:200 dilution, for 1 h at room temperature in the dark. The cells were washed 3 times, 5 min each after the incubation. The nuclei of the cells were counterstained with 150 nM of 4,6-diamidino-2-phenylindole (DAPI) for 5 min. After washing the cells thrice with PBS, the coverslips with cells were mounted with SlowFade® Gold antifade reagent (Molecular Probes, CA, USA) before sealing with transparent nail polish. Images were subsequently visualized and captured using Axioplan 2 imaging fluorescence microscope (Carl Zeiss) (43).

### RNA Extraction

Cells were collected after trypsinization and pelleted by centrifuging at 200 g for 5 min at 4°C. The resulting pellet was then washed twice with ice-cold 1X PBS (pH 7.4) and the supernatant aspirated completely after another round of centrifugation. Purification of total RNA was then carried out using RNeasy Mini kit (Qiagen, Hilden, Germany) according to the manufacturer's protocol. Up to 1 × 10^7^ cells, depending on the cell line, were disrupted in Buffer RLT (10 μl β-mercaptoethanol per 1 ml Buffer RLT was added before use) and homogenized using needle and syringe. One volume of 70% ethanol was added to the homogenized lysate, creating conditions that promote selective binding of RNA to the RNeasy membrane. The sample was then loaded to the RNeasy Mini spin column. Total RNA was bound to the membrane and contaminants were efficiently washed away with buffers RW1 then RPE. Lastly, high-quality RNA was eluted in 30–50 μl of RNase-free water. All binding, washing, and elution steps were performed by centrifugation in a microcentrifuge. Purified RNA was eluted and its concentration was determined using Nanodrop Spectrophotometer ND-1000 (Thermo Fisher Scientific, Lafayette, CO, USA).

### Reverse Transcription Polymerase Chain Reaction (RT-PCR) Analysis

Briefly, 3–5 μg of purified RNA template was used for cDNA synthesis using RT-PCR System kit (Promega, WI, USA). Reverse transcription was performed at 42°C for 1 h, followed by 65°C for 15 min. cDNA synthesis was completed after inactivation of transcriptase by incubation at 95°C for 5 min. cDNA was either stored at 4°C or on ice for immediate analysis or stored at −20°C until use. PCR amplification was carried out as shown in Table 2.10 using Thermal Cycler GeneAmp®PCR System 9600 (Applied Biosystems, CA, USA). Depending on the different primers used, the annealing temperature and number of cycles were optimized accordingly. The sequence of forward (F) and reveres (R) primers used for RT-PCR are as follows:

NCoR
F: 5′-TACCGCAGGAGCCATACAAGA-3′
R: 5′-GCTCAGTTGTGCTTTGGGAGC-3′
HBX
F: 5′-GTACTGCCAACTGGATCCTTC-3′
R: 5′-CCTCCCAGTCCTTAAACACAC-3′
B2M
F: 5′-ATCCAGCGTACTCCAAAGAT-3′
R: 5′-TTACATGTCTCGATCCCACT-3.′

### siRNA Mediated NCoR Knockdown and Cell Growth and Viability Assay

The NCoR siRNA was used to specifically knockdown the NCoR gene in HCC cells. A scrambled siRNA targeting the luciferase sequence which is not present in the mammalian genome was used as control siRNA. Cells were seeded in a similar way as mentioned earlier. Roughly 18–22 h after seeding, cells reaching about 60% confluency were transfected with either scrambled siRNA or NCoR siRNA. The transfection mixture was prepared by first mixing DMEM containing the appropriate concentration of siRNA with DMEM containing LipofectAMINETM 2000 (Invitrogen, CA, USA). This mixture was then incubated at room temperature for 20 min before adding to the respective seeding media. The transfected cells were returned to the CO2 incubator at 37°C and the morphology of transfected cells was analyzed under light microscope at the interval of 24–72 h. The efficacy of NCoR knockdown was analyzed by reverse transcription-polymerase chain reaction (RT-PCR). HBX positive and negative HCC cells treated with scrambled or NCoR siRNA were cultivated for 48 h in DMEM medium supplemented with 10% FBS and the viability of cells was determined by trypan blue exclusion test performed in triplicate. The cell viability was calculated as the percentage of viable cells divided by the total number of cells (43). The NCoR and the control siRNA sequences used in the assay are as follows:

N-CoR: 5′-AATGCTACTTCTCGAGGAAACA-3′
Luciferase: 5′-CGTACGCGGAATACTTCGA-3.′

## Data Availability Statement

All datasets generated for this study are included in the article/supplementary material.

## Author Contributions

ST performed the experiments, analyzed the data, and wrote the draft. SV performed experiments and revised the manuscript. RA provided clinical samples. MK conceived, designed and supervised the study, secured the funding, and wrote the manuscript.

### Conflict of Interest

The authors declare that the research was conducted in the absence of any commercial or financial relationships that could be construed as a potential conflict of interest.
